# Primer-Dependent and Primer-Independent Initiation of Double Stranded RNA Synthesis by Purified *Arabidopsis* RNA-Dependent RNA Polymerases RDR2 and RDR6

**DOI:** 10.1371/journal.pone.0120100

**Published:** 2015-03-20

**Authors:** Anthony Devert, Nicolas Fabre, Maïna Floris, Bruno Canard, Christophe Robaglia, Patrice Crété

**Affiliations:** 1 Aix-Marseille Université, Laboratoire de Génétique et Biophysique des Plantes, Marseille, France; 2 Centre National de la Recherche Scientifique, UMR 7265, Biologie Végétale et Microbiologie Environnementale, Marseille, France; 3 Commissariat à l’Énergie Atomique, Département des Sciences du Vivant, Institut de Biologie Environnementale et Biotechnologies, Marseille, France; 4 Aix-Marseille Université, AFMB UMR 7257, Marseille, France; 5 CNRS, AFMB UMR 7257, Marseille, France; University of Basel, SWITZERLAND

## Abstract

Cellular RNA-dependent RNA polymerases (RDRs) are fundamental components of RNA silencing in plants and many other eukaryotes. In *Arabidopsis thaliana* genetic studies have demonstrated that RDR2 and RDR6 are involved in the synthesis of double stranded RNA (dsRNA) from single stranded RNA (ssRNA) targeted by RNA silencing. The dsRNA is subsequently cleaved by the ribonuclease DICER-like into secondary small interfering RNAs (siRNAs) that reinforce and/or maintain the silenced state of the target RNA. Models of RNA silencing propose that RDRs could use primer-independent and primer-dependent initiation to generate dsRNA from a transcript targeted by primary siRNA or microRNA (miRNA). However, the biochemical activities of RDR proteins are still partly understood. Here, we obtained active recombinant RDR2 and RDR6 in a purified form. We demonstrate that RDR2 and RDR6 have primer-independent and primer-dependent RNA polymerase activities with different efficiencies. We further show that RDR2 and RDR6 can initiate dsRNA synthesis either by elongation of 21- to 24- nucleotides RNAs hybridized to complementary RNA template or by elongation of self-primed RNA template. These findings provide new insights into our understanding of the molecular mechanisms of RNA silencing in plants.

## Introduction

RNA-dependent RNA polymerases (RDRs) are required in RNA silencing, a conserved RNA-based mechanism involved in stability, protection, inheritance and expression of eukaryotic genomes at transcriptional or post-transcriptional levels [1–6]. RDRs produce long double-stranded RNAs (dsRNAs) that are subsequently cleaved by Dicer-like (DCL) nucleases into small interfering RNAs (siRNAs) ranging from 21 to 24 nucleotides (nt) [1–5]. siRNAs are then loaded onto ARGONAUTE (AGO) proteins that are part of the RNA silencing complex. One strand of the siRNA duplex then guides the ARGONAUTE complex to transcripts with complementary sequences, triggering transcript cleavage or the inhibition of translation [2–4,6]. ARGONAUTE complexes can also target genomic loci in a process known as RNA directed DNA methylation (RdDM) that promotes transcriptional gene silencing (TGS) [2–6].

In *Arabidopsis thaliana*, six RDRs have been identified. RDR1, RDR2 and RDR6 are functional in several siRNAs pathways whereas the functions of RDR3a, RDR3b and RDR3c, which belong to a distinct phylogenetic clade, have yet to be identified [1,4,5]. RDR2 synthesizes dsRNA from single stranded RNAs (ssRNAs) produced by the plant-specific RNA polymerase IV. RDR2-dependent dsRNA are then cleaved by DCL3 to produce the abundant 24-nt siRNAs that target heterochromatic and repetitive regions of the genome for direct sequence-specific DNA methylation and histone modification that leads to TGS [3–5,7–9]. RDR6 has the broadest range of substrates and is involved in the biogenesis of viral siRNAs [10], siRNAs from aberrant RNA transgenes during post-transcriptional gene silencing (PTGS) [11], tasiRNAs [12–15] and natsiRNA [16]. RDR6-dependent dsRNA are then cleaved by DCL4 or DCL2 to produce 21- or 22-nt long siRNA respectively [1–3,5]. RDR6 and RDR2 are also involved in PTGS transitivity and TGS transitivity, respectively. Transitivity is a phenomenon that leads to the production of secondary siRNAs upstream and downstream of the primary siRNA or miRNA binding site on a target transcript [1,17–23]. The production of secondary siRNAs allows the amplification of the RNA silencing effects [1,17–23]. However, it is not well understood how RDR6 and RDR2 are recruited to their RNA templates during transitivity. The cleavage of a transcript targeted by an miRNA produced from a miRNA:miRNA* duplex that contains asymmetrically positioned bulged bases is considered sufficient for the initiation of RDR6-dependent transitivity [17,23]. It is also unclear how RDR6 and RDR2 initiate complementary RNA synthesis during the amplification of RNA silencing. Initiation of RNA synthesis could occur as a primer-independent (*de novo*) process or a primer-dependent process using primary siRNA or miRNA as the primers for elongation [1,4,5]. Most known RNA polymerases rely on a primer-independent initiation mechanism, with only a few exceptions where short oligonucleotides or proteins are used as primers [4]. For *Arabidopsis* RDR6 there is *in vivo* evidence suggesting that both modes of initiation are used for complementary RNA synthesis [20,24,25]. RDR2 mediated production of nuclear secondary siRNAs is essential for RdDM during TGS in *Arabidopsis* and requires primary siRNAs [1,19]. However, the precise mechanism of RNA synthesis initiation was not determined.

I*n vitro* studies on purified RDRs from tomato leaves and on wheat germ extract have detected both primer-dependent and primer-independent activities [26,27]. Tomato RDR shares significant homology with all plant RDRs [4,28] therefore suggesting that primer-dependent RNA polymerase activity might be an intrinsic property of plant RDRs. However, while *in vitro* primer-independent (*de novo*) RNA polymerase activity has been observed for RDR6, no *in vitro* priming activity was detected [29,30]. RDR2 activity was studied in combination with PolIV on immunoprecipitates from *Arabidopsis thaliana* [31]. This shows that in absence of PolIV, RDR2 has no polymerase activity. In combination with active site mutated PolIV, RDR2 can synthesize short RNA in the absence of a primer but did not synthetize dsRNA by elongation of smRNAs hybridized to complementary RNA template [32].

Here we report on the *in vitro* enzymatic activities of purified recombinant RDR2 and RDR6. We demonstrate that RDR2 and RDR6 display primer-independent (*de novo*) RNA polymerase activity. We show that RDR2 and RDR6 can both elongate self-primed RNA templates. In addition, we have found that RDR2 and RDR6 can synthesize dsRNA by elongation of smRNAs hybridized to complementary RNA template. Our results demonstrate that the primed initiation mode is an intrinsic property of *Arabidopsis* RDR2 and RDR6 and that RDR6 priming activity is more sensitive to the *in vitro* experimental conditions and/or the absence of cellular co-factors than RDR2. These findings provide important new insights into our understanding of the molecular mechanisms of RNA silencing in *Arabidopsis*.

## Materials and Methods

### Plant material and transformation

*Arabidopsis thaliana* (Columbia ecotype) plants and *N*. *benthamiana* plants were grown on soil at 22°C in day/night cycle (12-h light/12-h dark) in growth rooms. *Arabidopsis* transgenic lines were obtained by *Agrobacterium*-mediated transformation using the floral dip procedure [32] as modified by Clough and Bent [33]. *Agrobacteria* containing the expression vectors pEarley100.*RDR2-HA* and pEarley100.*RDR6-HA* were used to complement *rdr2–2* and *rdr6–11* mutant plants, respectively. Transformants (named *RDR-HAcomp*) were selected on soil sprayed with a 150mg/L Basta (Glufosinate ammonium) solution. For phenotypic analysis, T2 plants were selected on soil and treated with Basta. Young flowers were harvested from six individual transgenic plants for pEarley100.*RDR2-HA* lines and pEarley100.*RDR6-HA* lines, quickly frozen in liquid nitrogen, and stored at −80°C for Northern blot analysis.

### Constructions of plasmids

The full-length cDNA of *RDR2* and *RDR6* were amplified from the ATG to the stop codon by PCR using primers AttB1-RDR2/AttB2-RDR2, and AttB1-RDR6/AttB2-RDR6 (S1 Table). The resulting amplicons were cloned into pDONR207 (Invitrogen) to give pDONR207-*RDR2* and pDONR207-*RDR6* plasmids. Amplification of *RDR2-HA* was realized on pDONR207-*RDR2* with primers Age1-RDR2 and SmaI-RDR2-2xHA. Amplification of *RDR6-HA* was realized on pDONR207-*RDR6* with primers Age1-RDR6 and XhoI-RDR6-2xHA. The *RDR6-HA* amplicon was then digested by AgeI/XhoI and cloned into pEAQ-HT plasmid [34] to obtain pEAQ-*RDR6-HA*. The *RDR2-HA* amplicon was then digested by AgeI/SmaI and cloned into pEAQ-HT plasmid to obtain pEAQ-*RDR2-HA*. Cloned RDR sequences were verified by DNA sequencing. *RDR* mutants were obtained by linear amplification of pDONR207-*RDR2* and pDONR207-*RDR6* plasmids with primers RDR2mutFW and RDR6mutFW respectively, to substitute by an Ala residue the putative catalytic Asp residues located at amino acid 834 of RDR2 and 867 of RDR6. Amplification products were then digested with DpnI to eliminate the plasmid templates and introduced into *E*. *coli*. Mutations were checked by DNA sequencing after plasmid purification of pDONR207-*RDR2m* and pDONR207-*RDR6m*. Amplification of *RDR2m-HA* was realized on pDONR207-*RDR2m* with primers Age1-RDR2 and SmaI-RDR2–2xHA and cloned into pEAQ-HT plasmid to obtain pEAQ-*RDR2m-HA*. Amplification of *RDR6m-HA* was realized on pDONR207-*RDR6m* with primers Age1-RDR6 and XhoI-RDR6-2xHA and cloned into pEAQ-HT plasmid to obtain pEAQ-*RDR6m-HA*. The full-length cDNA of *RDR2* and *RDR6* with 2xHA tag in 3’ end were amplified by PCR using primers AttB1-RDR2/AttB2-2xHA and AttB1-RDR6/ AttB2-2xHA with pEAQ-*RDR2-HA* and pEAQ-*RDR6-HA* templates, respectively. The resulting amplicons were cloned into pDONR207 (Invitrogen) to give pDONR207-*RDR2-HA* and pDONR207-*RDR6-HA* plasmids. A LR recombinant reaction was carried out between the expression vector pEARLEY100 [35] and either pDONR207-*RDR2-HA* or pDONR207-*RDR6-HA* to obtain pEarley100.*RDR2-HA* and pEarley100.*RDR6-HA* plasmids used for *rdr* mutant complementation.

### Expression, purification of recombinant RDR proteins and immuno blotting

Transient expression of recombinant RDRs using *agrobacterium* containing either pEAQ-*RDR2-HA*, pEAQ-*RDR6-HA*, pEAQ-*RDR2m-HA* or pEAQ-*RDR6m-HA* plasmids has been realized in *N*. *benthamiana* according to the protocol established by Voinnet *et al*. [36]. Infected leaves were collected after 60h of incubation, frozen in nitrogen and conserved at −80°C. Protein extraction and purification were done according to Curaba and Chen [29] with some modifications. Typically, 5g of harvested infiltrated tissues were ground in a mortar. One volume of tissue powder was mixed with two volumes of extraction buffer (50 mM Tris-HCl, pH 7.6, 150 mM NaCl, 5 mM MgCl2, 0.1% (v/v) triton, 10% (v/v) glycerol, 1mM phenylmethylsulfonyl fluoride, 0.05% (v/v) 2-mercaptoethanol, 0.5% (v/v) Polyvinylpyrrolidone (PVP)) and one protease inhibitor mixture tablet per 20 ml (Roche), and incubated for 20 min at 4°C. Lysate was centrifuged 10 min at 1,500 g at 4°C, then for 30 min at 20,000 g at 4°C and the supernatant was collected (when necessary, additional centrifugation steps were performed to eliminate dark pigments). 200 μl of anti-HA affinity matrix (Roche) were stacked in a chromatography column and washed 3 times with 5ml of extraction buffer. The supernatant was passed through the column 2 times. The beads were washed 3 to 4 times with 20 volumes of the washing buffer (20 mM Tris-HCl, pH 7.6, 100 mM NaCl, 0.1 mM EDTA, 0.05% (v/v) Tween 20, 0.05% (v/v) 2-mercaptoethanol and one protease inhibitor tablet/20 ml) and finally resuspended in 3 volumes of storage buffer (20 mM HEPES- KOH, pH 7.6, 20 mM NaCl, 0.2 mM EDTA, 20% (v/v) glycerol, 0.05% (v/v) 2-mercaptoethanol and one protease inhibitor tablet/20 ml), and stored in aliquots at −80°C. RDR-HA-bound on anti-HA-agarose beads were analyzed on a 4–20% polyacrylamide gradient-SDS gel and transferred to Protran BA 85 Nitrocellulose membrane (Whatman). Monoclonal anti-HA antibody (Sigma) were used as primary antibody, and Sheep Anti-Mouse IgG ECL Antibody, HRP Conjugated (GE Healthcare) as secondary antibody. ECL Plus Western Blotting Detection Reagents (GE Healthcare) and Fluorescent Image analyser FLA-5000 (Fuji) were used for detection and image acquisition.

### Preparation of RNA duplexes

Chemically synthesized and PAGE purified heteropolymeric single-stranded RNA (ssRNA) templates were obtained from Dharmacon (S2 Table). In a 20μL reaction assay containing 500 mM of NaCl, 150 pmol of RNA37 was mixed with either 100 pmol of 5’[32P]-labelled RNA21, RNA22 or RNA24. The mixture was heated at 95°C for 2 min and slowly cooled for 3h at room temperature. Water was added to the mixture up to 50 μl before purification on G25 Sephadex columns (Health care) to remove NaCl and free [γ-32P] ATP.

### Polymerase activity assays

RDR activity assays were performed according to Makeyev and Bamford [37] with some modifications. Assays were conducted in 20μl reaction mixtures assembled on ice, containing 50 mM HEPES-KOH (pH 7.6), 10 mM MgCl2, 0.1 mM EDTA, 100 μM each of cold ATP, CTP, GTP and UTP, 100 nM of single-stranded or partially double-stranded RNA. In some experiments RNA templates were labeled by 5’ phosphorylation using [γ-32P]ATP (3000 Ci/mmol) and T4 kinase (Promega). In experiments with unlabeled templates, 150 nM of [α-32P]GTP (3000 Ci/mmol) were added to the reaction mixtures. To start the reaction, about 2.2 pmol of RDR bound on anti-HA-agarose beads were added and tubes were incubated at 25°C for 4h in a thermomixer set for a gently 30s-long agitation (300 rpm) every 10 min. After incubation, reactions were placed on ice and 4 μl of polymerisation products were either directly mixed with a formamide/EDTA gel-loading buffer or treated with RNase T1 before addition of loading buffer (see *Nuclease Treatments*). Polymerisation products were then heated 5 min at 65°C and loaded on a 14% acrylamide-bisacrylamide [19:1], 7 M urea gels, using TTE buffer (89 mM Tris/HCl (pH 8.0), 28 mM Taurine and 0.5 mM EDTA). The gels were exposed and analysed using photo-stimulated plates and a Fluorescent Image Analyzer (Fluorescent Image Analyser FLA3000, Fuji).

### Nuclease treatments

Four microliters of the polymerase reaction mixture supplemented with 5 units of RNaseT1 (Invitrogen) were incubated 30 min at 30°C and the reaction was placed on ice. Then, 1μl of 200 mM Dithiothreitol in addition to the formamide/EDTA loading buffer was added to the mixture and samples were heated 5 min at 65°C before loading on polyacrylamide/Urea gels.

## Results

### Production of functional RDR2 and RDR6 with a double C-terminal HA-tag

**Fig 1:**
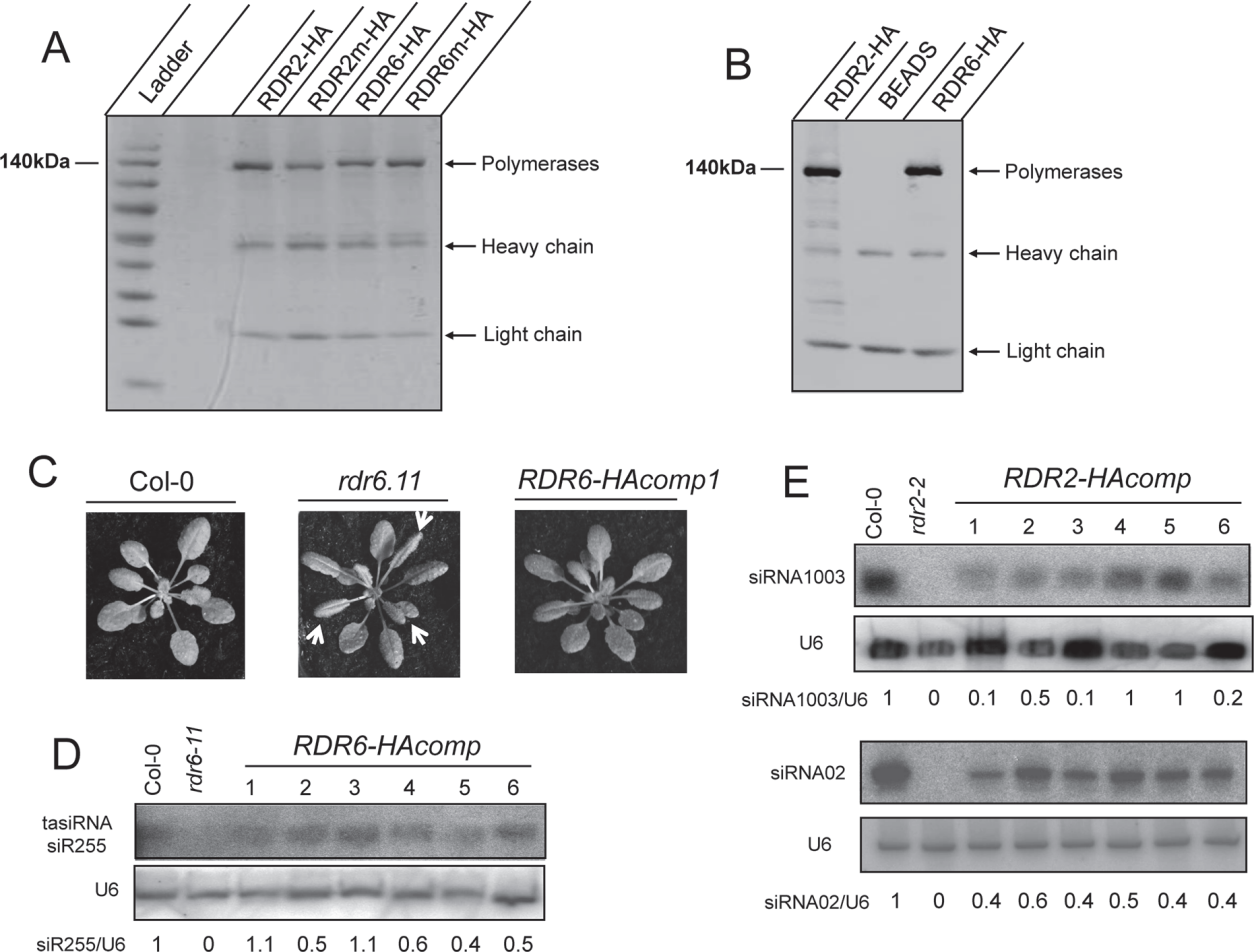
Purification of RDR-HA and RDRm-HA and complementation of *rdr2–2* and *rdr6–11* mutations by RDR2-HA and RDR6-HA. (A) Coomassie blue stained protein gel of immunopurified RDRs. After immunoprecipitation, 2.5μl of HA-conjugated beads, corresponding to 250ng of proteins, were loaded on a 4–20% polyacrylamide gradient-SDS gel. Heavy and light chains from HA antibody are visible. (B) Western blot performed with anti-HA antibodies. After immunoprecipitation, 1μl of HA-conjugated beads were loaded on a 4–20% polyacrylamide gradient-SDS gel. The secondary antibody cross reacts with the heavy and light chains of the HA antibody. (*C*) 24-day-old Columbia wild type (*Col-0*) plant, *rdr6–11* mutant and one *rdr6–11* transgenic line containing the 35S::RDR6-HA transgene (*RDR6-HAcomp1*). The downward curling rosette leaf phenotype of *rdr6–11* mutants (arrows) was rescued by RDR6-HA expression. (*D*) RNA blot analysis of the accumulation of the *TAS1*-derived tasiRNA siR255. The analysis was performed on *rdr6–11* mutant and six independent 35S::RDR6-HA *rdr6–11* transgenic lines. (E) RNA blot analysis of the accumulation of the heterochromatic siRNA1003 and siRNA02. The analysis was performed on *rdr2–2* mutant and six independent 35S::RDR2-HA *rdr2–2* transgenic lines (*RDR2-HAcomp*). U6 snRNA hybridization served as control for total RNA loading and normalized values with Col-0 control set to 1 are indicated.

To produce RDR2 and RDR6 we chose a transient expression system in *N*. *benthamiana*. Full-length RDR2 and RDR6 coding sequences with a double HA tag at the 3’ end were cloned into the pEAQ-HT plasmid. pEAQ-HT contains a gene for the expression of the P19 inhibitor of RNA silencing *in planta* and the introduced coding sequences are flanked by the 5’-untranslated region (UTR) and the 3’-UTR from Cowpea mosaic virus (CPMV) RNA-2. These features allow for an extremely high-level production of proteins [34]. Tagged RDR2 and RDR6 (RDR2-HA and RDR6-HA) expressed in *N*. *benthamiana* leaves were immunopurified with HA-conjugated beads and the purity of the enzymes was checked on polyacrylamide gel stained with coomassie blue and by western blot analysis showing that in each case a single polypeptide was purified (Fig. 1A and 1B). To ensure that RDR activities observed in polymerase assays were not due to contaminating polymerases, we also produced mutated versions of RDR2-HA (RDR2m-HA) and RDR6-HA (RDR6m-HA) where a catalytic aspartate residue critical for RDR activity is substituted by alanine [29, 37, 38]. We obtained about 100 *n*g of purified RDR per 1μl of HA-conjugated beads (Fig. 1A). The functionality of the HA-tagged RDR6 and RDR2 proteins was confirmed by stable expression under the control of the cauliflower mosaic virus (CaMV) 35S promoter in *A*. *thaliana rdr6*.*11* and *rdr2*.*2* mutants respectively. The *rdr6*.*11* mutant has distinctive downward curling of the rosette leaves. Expression of RDR6-HA fully restored the wild-type phenotype and the production of an RDR6-dependent tasiRNA (siR255) (Fig. 1C and 1D). *rdr2*.*2* mutant does not show an obvious visible phenotype, but is impaired in the production of some endogenous 24nt siRNAs. Northern blot analysis showed that expression of RDR2-HA restored the production of these siRNAs (siRNA1003, siRNA02) (Fig. 1E).

### RDR2-HA and RDR6-HA initiate *in vitro* RNA synthesis using a primer-independent (*de novo*) mechanism

**Fig 2:**
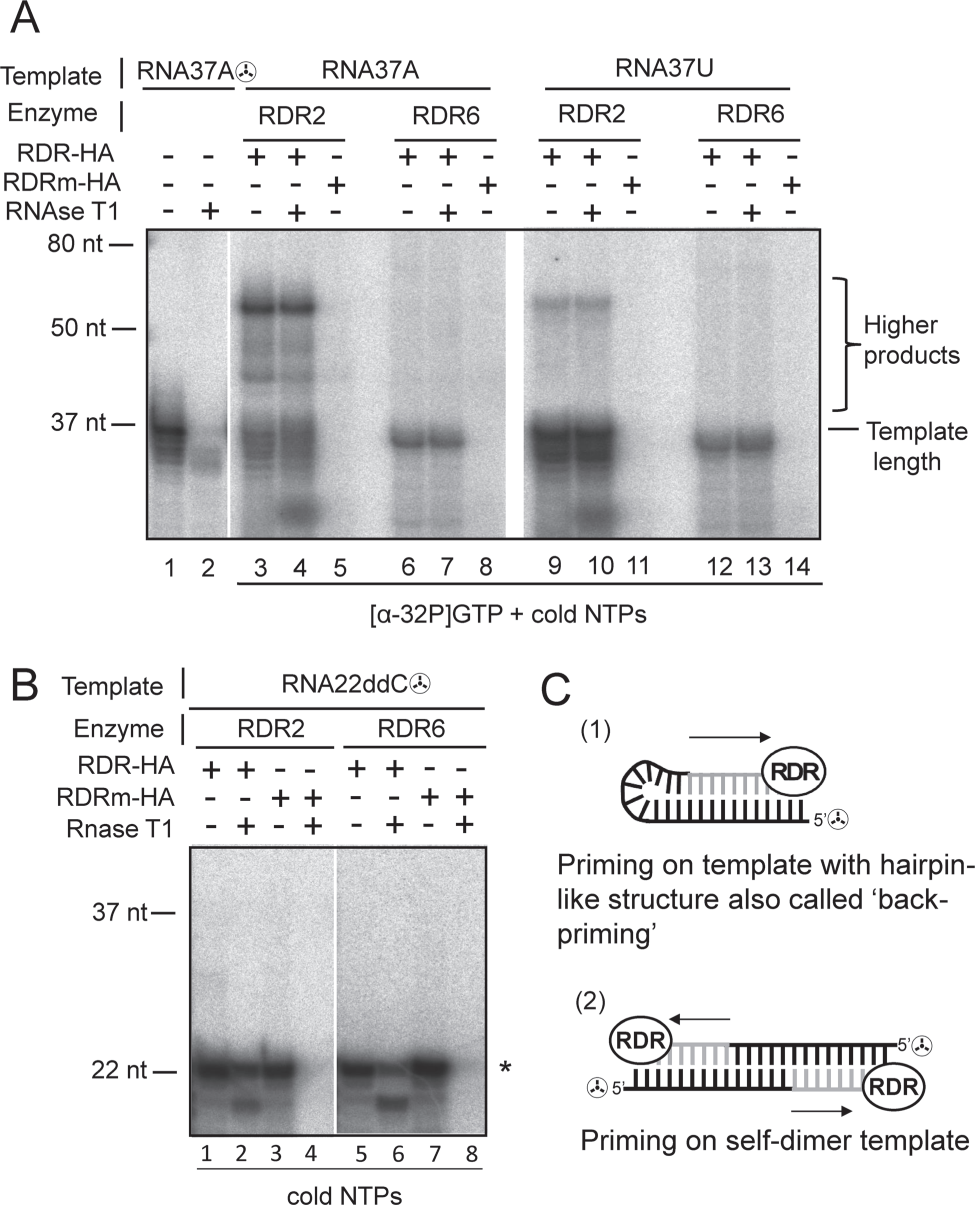
RDR2-HA and RDR6-HA initiate *in vitro* RNA synthesis using a *de novo* (primer-independent) mechanism. (A) RNA polymerase assays of purified RDR2-HA and RDR6-HA on 37nt long ssRNA templates (RNA37A and RNA37U) in presence of cold NTPs and trace amount of [α32-GTP]. lane1: 5'[32P]-labeled RNA37A (RNA37A☢) is used as a size marker. lane2 RNA37A☢ treated with RNAse T1 as a control for RNAseT1 digestion in conditions that almost completely degrade the ssRNA template (B) Purified RDR2-HA and RDR6-HA were incubated with cold NTPs and a 22-nt 5’[32P]-labeled ssRNA template blocked at its 3′ end by dideoxycytidine (RNA22ddC☢). (*) A single star indicates the position of the 5’[32P]-labeled template. (C) Schematic representation of mechanisms that allow RNA polymerases to generate products higher than the RNA template size (“higher products”). Arrows indicate the direction of RNA polymerization. Template RNA in black, synthesized RNA by RDR in grey.

RNA polymerase activity assays for RDR2-HA and RDR6-HA were performed using two short chemically synthesized and PAGE purified heteropolymeric ssRNA templates with a 37 nt random sequence containing a monophosphate at the 5’ terminus, a feature of small RNAs generated by DICER-like enzymes (S2 Table). The recombinant RDRs and cold ssRNA templates (RNA37A and RNA37U) were incubated in the presence of all four cold ribonucleoside triphosphates (NTPs) and trace amounts of [α-32P] GTP (Fig. 2A). For RDR2-HA and RDR6-HA analysis of reaction products on a denaturing polyacrylamide gel showed the production of labeled RNA of various sizes (Fig. 2A lanes 3,6,9,12). No labeling was detected using the mutated RDRs (Fig. 2A lanes 5,8,11,14). The band-product pattern is strikingly different between RDR2-HA to RDR6-HA: the former generates labeled products longer than the template and a major product nearly up to twice the size of the template (referred thereafter as “higher products”) (Fig. 2A lanes 3 and 9), whereas for RDR6-HA the major product is of apparent template size (Fig. 2A lanes 6 and 12). To show that the RDR2-HA and RDR6-HA exhibit authentic polymerase activity leading to synthesis of RNA complementary to the ssRNA template and resulting in production of dsRNA, polymerase products were treated with RNAse T1. RNAse T1 digests ssRNA, but not dsRNA, by cleaving between guanosine 3'-phosphate residues and the 5'-OH residues of adjacent nucleotides. In this experiment 5'[32P]-labeled RNA37A was used as a size marker (Fig. 2A lane1) and 5'[32P]-labeled RNA37A treated with RNAse T1 was used as a control for RNAseT1 digestion (Fig. 2A lane 2). Without prior polymerase action the 5’[32P] labeled RNA37A ssRNA template is degraded by RNAse T1 (Fig. 2A compare lane 1 and 2) whereas polymerase products are resistant to RNase T1 (Fig. 2A lanes 4,7,10,13) thus confirming their double-stranded nature. Similar results are obtained when RNA polymerase assays with RDR2-HA and RDR6-HA are performed using 5’[32P]-labeled ssRNA37 template and cold NTPs (S1 Fig.). RDR2-HA is able to synthesize “higher products”, whereas RDR6-HA is not. However, we note that RNaseT1 treatment of RDR6-HA generated products only partially removes the radiolabeled input template, indicating that the RDR6 products are dsRNA (S2 Fig. lane 5). To establish the nature of the “higher products” we performed polymerase assays using a 5’[32P]-labeled RNA template blocked at its 3′ end by a dideoxycytidine (RNA22ddC) (S2 Table). In this case, both polymerases produced the same banding pattern with the longer product at template size and without the “higher products” (Fig. 2B). The absence of template elongation by the 3' ddC nucleotide shows that the "higher products" produced by RDR2-HA are the result of RNA synthesis starting at the 3’ end by either self-priming or intermolecular priming mechanism (Fig. 2C). Furthermore, this experiment demonstrates that the two polymerases are able to initiate RNA synthesis *de novo* in the absence of self-priming. For both enzymes, we could also observe RNAse T1 resistant products that were smaller than the template length (Fig. 2B lanes 2 and 6) indicating that *de novo* synthesis may start internally on the provided template. Altogether, these results show that both RDR2-HA and RDR6-HA are active RNA polymerases that are able to initiate RNA synthesis using a primer-independent (*de novo*) mechanism. In addition, RDR2-HA is able to initiate RNA synthesis using a self-priming mechanism.

### RDR2-HA and RDR6-HA initiate *in vitro* RNA synthesis using a primer-dependent initiation mechanism

**Fig 3:**
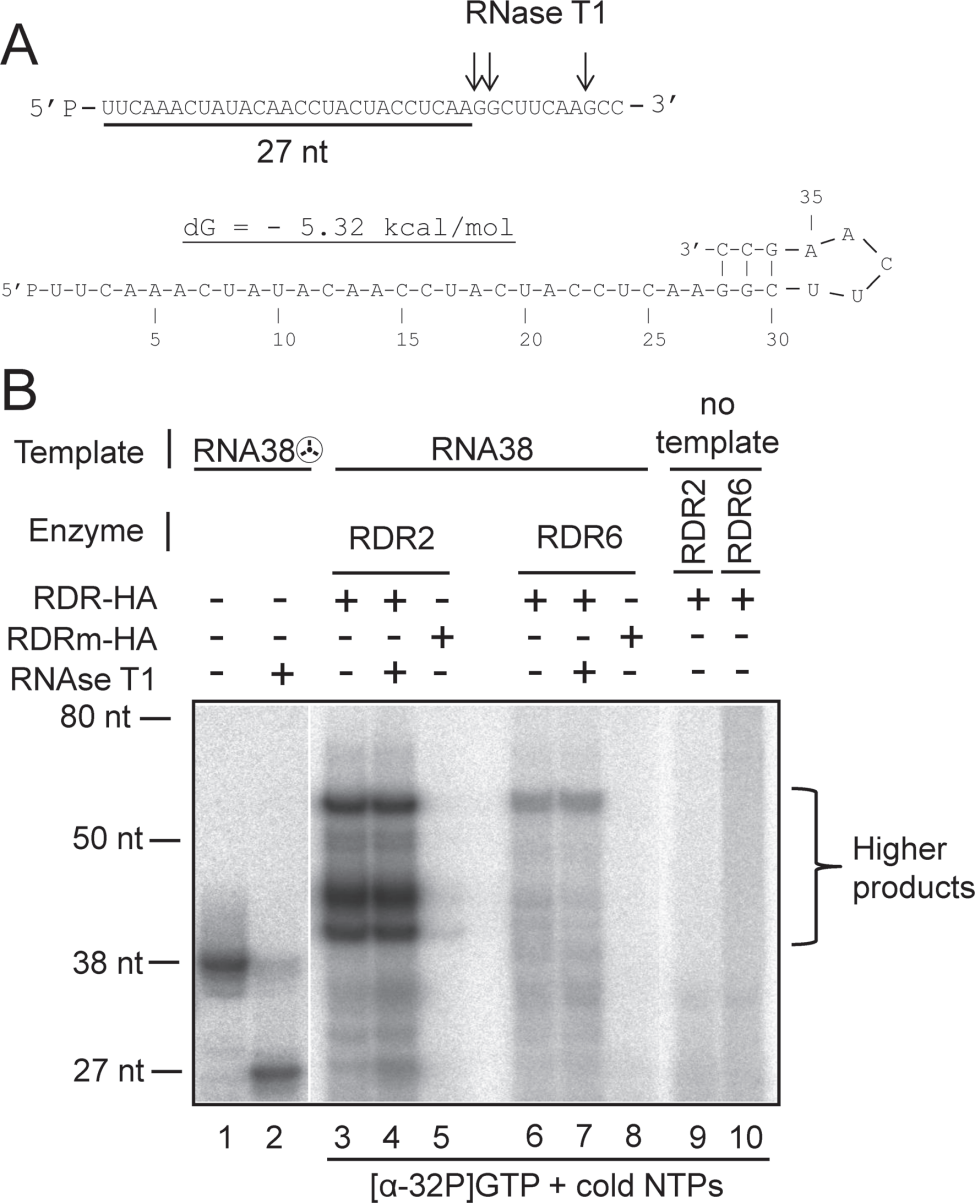
Polymerisation activity of RDR2-HA and RDR6-HA on an ssRNA template designed to adopt a stable hairpin-like conformation in the 3' terminus region. (A) Sequence and RNAse T1 cleavage sites of the 38-nt-long ssRNA38 template and schematic representation of the energetically most-favorable predicted secondary structure of ssRNA38 template using OligoAnalyser 3.1 (IDT). (B) Purified RDR2-HA and RDR6-HA were incubated with RNA38 template, cold NTPs and trace amounts of [α32-GTP]. 5'[32P]-labeled ssRNA38 is used as a size marker (RNA38A☢) (lane1) and as a control for RNAseT1 digestion (lane2).

In polymerase assays using non labeled ssRNA template, cold NTPs and trace amount of [α-32P]GTP, RDR2-HA was able to synthesize “higher product” (Fig. 2A). Similar results were obtained when RDR2-HA was incubated with 5’[32P]-labeled ssRNA37 templates and cold NTPs. In these conditions detection of “higher products” is only possible if there are generated by elongation of the radiolabeled input template (S1 Fig. lanes 1 and 2). Therefore the “higher products” generated by RDR2-HA are likely due to a priming activity that corresponds to self-primed elongation of the ssRNA template from its 3'-terminal hydroxyl by intramolecular (Fig. 2C.1) or intermolecular template interactions (Fig. 2C.2). This hypothesis is also supported by our assays with a ddC blocked template (Fig. 2B). RDR6-HA did not perform self-primed elongation of RNA37A and RNA37U templates (Fig. 2A and S1 Fig.). As RDR6 is a very close paralog of RDR2 [4] we hypothesized that they share catalytic properties and that experimental conditions or template features could account for the difference in the priming efficiency between RDR2-HA and RDR6-HA. As 3’ terminal secondary structures can promote primer-dependent initiation [39] we designed an ssRNA template that can adopt a stable hairpin-like conformation in the 3' terminus region (named RNA38, Fig. 3A) and performed RNA polymerase assays using this template, cold NTPs and trace amounts of [α-32P]GTP. Under these conditions both RDR2-HA and RDR6-HA produce “higher products” resistant to RNAase T1 (Fig. 3B, lanes 3,4,6,7). To confirm that the “higher products” observed were due to a self-primed elongation of the RNA38 template, we incubated each RDR with 5’[32P]-labeled RNA38 template and cold NTPs. In these conditions, we observe a product pattern similar to that observed with labeled RNA38 and [α-32P]GTP (Fig. 3B and S2 Fig.). The “higher products” were double-stranded, as shown by the RNase T1 resistance assay (Fig. 3B lanes 4 and 7, S2 Fig. lanes 2 and 5). Unlike RDR6-HA, RDR2-HA generates from RNA37 and RNA38 templates, clearly detectable primer-dependent products of various sizes (Fig. 2A lanes 3,4,9,10, 3B lanes 3 and 4, S1 Fig. lanes 1 and 2, S2 Fig. lanes 1 and 2).

This size heterogeneity observed for RDR2-HA products could arise from a more efficient transcription initiation of RNA templates with 3’ terminal nucleotide primed at different positions. Another nonexclusive hypothesis could be that RDR2-HA start transcription initiation of RNA templates with 3’ terminal nucleotide primed at a unique position but has reduced elongation processivity which gives rise to abortive products.

We conclude that both RDR2-HA and RDR6-HA are able to perform elongation of self-primed ssRNA templates. However, contrary to RDR2-HA, RDR6-HA has elongated only one of the three RNA templates used, RNA38. This could be due to different nucleotide sequences or to the nature of the 3’ terminal nucleotide of the templates (S2 Table). In addition, RNA38 molecules can be self-primed with more stable secondary structures than RNA37A and RNA37U, this could allow RDR6-HA to perform self-primed elongation of RNA38.

### RDR2-HA and RDR6-HA initiate *in vitro* dsRNA synthesis by elongation of 21-, 22- and 24- nt small RNAs hybridized to a complementary RNA template

**Fig 4:**
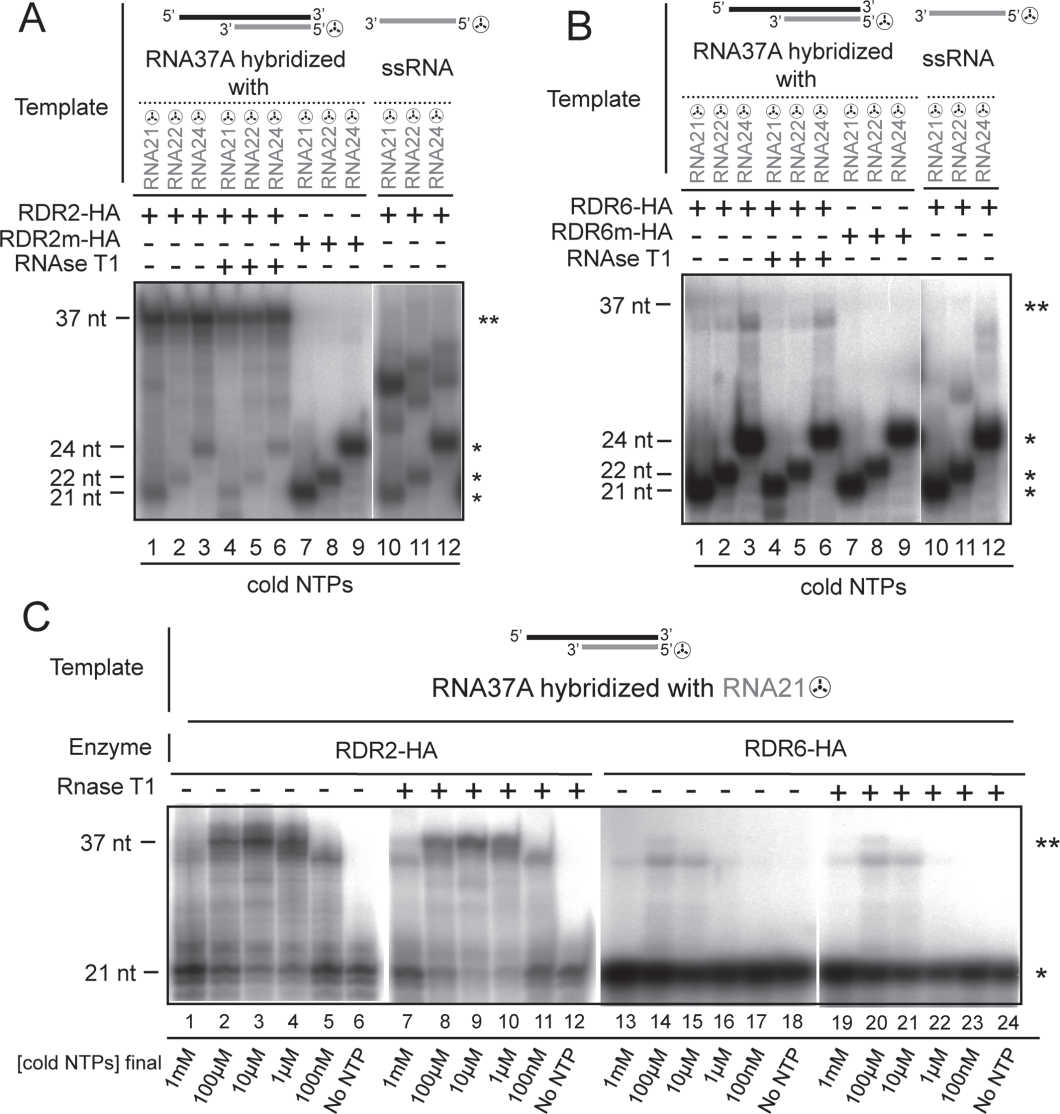
Polymerisation activity of RDR2-HA and RDR6-HA on partial dsRNA templates. (A) Purified RDR2-HA and RDR2m-HA were incubated with partial dsRNAs (S3 Fig.) formed with RNA37 hybridized with either 5’[32P]-labeled 21-, 22- or 24-nt ssRNA (RNA21☢, RNA22☢, RNA24☢) (lanes 1–9). Purified RDR2-HA was incubated with either RNA21☢, RNA22☢, RNA24☢ oligonucleotides (lanes 10–12). (B) Purified RDR6-HA and RDR6m-HA were incubated with partial dsRNAs formed with RNA37 hybridized with either RNA21☢, RNA22☢ or RNA24☢ oligonucleotides (lanes 1–9). Purified RDR6-HA was incubated with RNA21☢, RNA22☢, RNA24☢ oligonucleotides (lanes 10–12). (C) Purified RDR2-HA and RDR6-HA were incubated with partial dsRNAs formed with RNA37A hybridized with 5’[32P]-labeled RNA21☢ oligonucleotide and with various concentrations of all four cold NTPs. (*) A single star indicates the position of the 5’[32P]-labeled ssRNA. (**) A double star indicates the position of the full-length elongated RNA template.

RNA silencing models propose that secondary siRNAs are produced by RDR2- and RDR6-mediated elongation of small RNAs (smRNA) i.e. primary siRNA or miRNA hybridized to target ssRNA [1,19]. *In vivo* studies have shown that AtRDR6 has primer elongation activity [20], this activity has not been demonstrated *in vitro* with RDR6 alone [29] or with SGS3, a dsRNA-binding protein that physically interacts with RDR6 [40] and that stabilizes RDR6 templates *in vivo* [15]. Therefore, our next challenge was to determine whether purified RDR6-HA and RDR2-HA were able to elongate smRNA hybridized to a complementary ssRNA template and to determine if this priming activity is dependent on smRNA size. 5’[32P]-labeled smRNAs of 21-, 22- and 24-nt corresponding in size to smRNAs involved in RNA silencing were used as primers by hybridization to the 3’ complementary region of a 37-nt ssRNA template (S3 Fig.). These partial dsRNA mimic the *in vivo* hybridization of an siRNA or miRNA to a target RNA. Each RDR was incubated with the 5’[32P]-labeled partial dsRNA templates and cold NTPs (Fig. 4). These experimental conditions only allow detection of products resulting in elongation of 5’[32P]-labeled smRNAs. For RDR2-HA, we obtained a strongly labeled RNase T1-resistant product of 37-nt size, which corresponds to synthesis of the full-length dsRNA by elongation from the 5’[32P]-labeled smRNAs (Fig. 4A lanes 1–6, 4C lanes 2–4 and 8–10, S4 Fig. lanes 3 and 4). The intensity of the 37-nt signal was similar, irrespective of the hybridized smRNA size (Fig. 4A lanes 1–6). No polymerisation was observed with mutated RDR2-HA (Fig. 4A lanes 7–9, S4 Fig. lane 5). To exclude the possibility that the 37-nt signal was produced by self-priming activity on non hybridized 5’[32P]-labeled smRNAs, reactions containing only the 5’[32P]-labeled smRNA were performed (Fig. 4A lanes 10–12). In this case, “higher products” corresponding to self-primed elongation of smRNAs were observed but no 37-nt product was detected. For RDR6-HA, we observed signals corresponding to dsRNA produced by smRNA elongation but the intensity of the signals was much weaker than that observed for RDR2-HA (Fig. 4B lanes 1–6, 4C lanes 13–15 and 19–21, S4 Fig. lanes 6 and 7). In addition, the main band was a few nucleotides below the template size indicating that RDR6-HA performed an incomplete synthesis of complementary strand. When each mutated RDR was incubated with partial dsRNA templates (i.e. no polymerase activity), a strong RNAse T1 resistant signal corresponding to the 5’[32P]-labeled smRNAs showed that in our experimental conditions, hybridization of the smRNAs to complementary RNA37 template was effective (Fig. 4A lanes 7–9, 4B lanes 7–9). Altogether, these results show that RDR2-HA and RDR6-HA are able to initiate *in vitro* dsRNA synthesis by elongation of smRNAs hybridized to complementary RNA template.

### *In vitro* primer-dependent RNA polymerisation by RDR6-HA and RDR2-HA is dependent on the concentration of NTPs

Two studies have reported *in vitro* primer-independent RNA polymerisation of recombinant RDR6 but no primer-dependent RNA polymerisation was detected [29,30]. Although weak, we showed a primer-dependent activity for RDR6-HA (Fig. 4B and 4C, S4 Fig.). One hypothesis is that *in vitro* primer-dependent activity of recombinant RDR6 could have been prevented by the N-terminal localisation of the HA tag in contrast to the C-terminally HA-tagged RDR6 used in our study. Another hypothesis is that primer-dependent RNA polymerisation is sensitive to differences in the polymerase reaction mixtures, such as NTP concentration that is known to modulate the ratio between priming and *de novo* activity of viral RDR [41]. To test this latter hypothesis we used RDR6-HA and RDR2-HA to perform 21-nt smRNA elongation assays on partial dsRNA template with different NTPs concentrations (Fig. 4C). No priming activity was detected for RDR6-HA at 1μM and 100nM cold NTPs. At 100μM and 10μM cold NTPs we observed a weak signal a few nucleotides below the 37 nt full length product (Fig. 4C lanes 14 and 15) indicating that RDR6-HA performed an incomplete primer elongation as observed above (Fig. 4B). At 1mM cold NTPs, the concentration used in previous studies on recombinant RDR6, the signal is at the limit of detection (Fig. 4C lane 13). Therefore NTP concentration strongly influences the efficiency of RDR6-HA primer-dependent activity and could explain why this activity was not detected by others [29,30]. For RDR2-HA, a signal corresponding to the synthesis of full-length dsRNA by elongation of the 5’[32P]-labeled smRNAs was detected at 100 μM, 10μM and 1 μM cold NTPs (Fig. 4C, lanes 2–4) and a signal corresponding to an incomplete smRNA elongation was detected at 1mM and 100nM cold NTPs (Fig. 4C lanes 1 and 5). As observed for all smRNAs tested at 100 μM (Fig. 4A and 4B), the efficiency of primer-dependent activity of RDR2-HA was much higher than for RDR6-HA at each NTP concentration tested (Fig. 4C). These results indicate that under *in vitro* conditions, priming activity is dependent on NTP concentration and that RDR2-HA is more efficient than RDR6-HA at elongating smRNA hybridized to a complementary RNA template. RDR6-HA might exhibit stronger primer-dependent activity in the presence of cellular factors or under other yet-to-be defined experimental conditions. Altogether, our results show that RDR2-HA and RDR6-HA are both potent primer-dependent and primer-independent RNA polymerases.

## Discussion

Our findings demonstrate that *in vitro* purified recombinant RDR2-HA, like RDR6-HA has an RNA dependent RNA polymerase activity. This is consistent with the involvement of RDR2 in the biogenesis of dsRNA that give rise to heterochromatic and repeat-associated siRNAs, the most abundant endogenous siRNAs in plants. [5,7–9]. RDR2-HA and RDR6-HA can initiate dsRNA synthesis *in vitro* by a primer-independent (*de novo*) mechanism (Fig. 2). These results are in agreement with recent *in vivo* studies on the role of RDR6 in the tasiRNA-generating pathway. It was shown that for the *TAS1, TAS2 and TAS3* ssRNA templates, RDR6-mediated synthesis of complementary RNA is initiated internally at the second, third or fourth nucleotide from the 3’ end, suggesting a *de novo* initiation mechanism for RDR6 polymerase activity *in vivo* [24,25].

We showed that RDR2-HA and RDR6-HA synthesized RNA products that were longer than the RNA template size and nearly twice the size of the template. These products are dsRNA and result from a self-primed elongation of the ssRNA template from its 3'-terminal hydroxyl. RDR6-HA could not perform *in vitro* self-primed elongation of all ssRNA templates tested but was active on an RNA template designed to adopt a stable hairpin-like conformation at the 3' terminus region. In contrast, RDR2-HA was able to efficiently extend from a RNA 3’-hydroxyl end at a variety of positions on an RNA template (Fig. 2 and Fig. 3). RDR2 and RDR6 share almost identical active site architecture with similar amino acids residues but have a different additional large N-terminal domain with uncharacterized function that could explain the difference in priming efficiency [4]. This difference in priming efficiency may reflect a requirement for cellular co-factors in RDR6 priming or other yet-to-be defined experimental conditions.

Does elongation of self-primed RNA template by RDRs have a functional significance in RNA silencing? Improperly terminated and unpolyadenylated mRNA (known as aberrant RNAs) from sense transgenes are targeted by RDR6-mediated RNA silencing in *Arabidopsis* [42]. In such cases mRNA with defective 3’-ends lack the full complement of RNA-binding proteins. The naked 3’ ends can then lead to the spontaneous formation of 3’ hairpin-like structures, allowing priming of dsRNA synthesis by RDR6. In support of this view, enhanced RDR6-dependent RNA silencing phenotypes have been observed in *Arabidopsis* mutants that are deficient for mRNA 3’-end formation [43]. RDR2 is involved in TGS and synthesizes dsRNA from RNAs produced by the plant-specific DNA-dependent RNA polymerase IV [6,31,44]. We observed efficient self-primed elongation of ssRNA template by RDR2 (Fig. 2 and 3). A recent study address the activity of RDR2 in combination with the plant-specific DNA-dependent RNA polymerase IV with which it interacts [31]. In this work, it is shown that RDR2 alone has no polymerase activity, while RDR2, in combination with inactive PolIV, can efficiently synthesize short RNA in the absence of a primer. In addition, this study did not show efficient elongation of a primed RNA hybridized to complementary RNA template by RDR2 in combination with inactive PolIV, while in this condition efficient primer independent synthesis from short RNA template is found. Several causes may explain the differences with our results. We purified RDR2 from overexpressing *N*. *Benthamiana* as a single polypeptide while in the study of Haag et al., it was immunopurified from *Arabidopsis* in combination with PolIV, therefore some *Arabidopsis* inhibitors of RDR2 polymerase activity may have copurified. For exemple, PolIV even inactive may inhibit RDR2 polymerase activity. However our results are still compatible with a model were selfed-primed PolIV transcripts are converted to double-stranded RNA by RDR2 activity to serve as DCL3 substrates. Double-stranded RNA synthesis from self-primed PolIV transcripts by RDR2 would however suppose that the 3’ end of nascent RNA be previously released from the PolIV transcribing complex.

*In vivo*, RDR2 priming-dependent activity could be involved in the production of dsRNA from unpolyadenylated RNAs that adopt 3’ hairpin-like structures, such as those produced by PolIV transcription of transposons, retroelements and other repetitive DNA elements [3,7–9,44]. In addition, the occurrence of hairpin-like structures at unpolyadenylated 3’ ends could be a feature that promotes the recruitment of RDR2 and RDR6 to their RNA substrates. In such cases, RDR recruitment and initiation of complementary RNA synthesis would be independent of primary siRNA or miRNA targeting. This hypothesis is supported by a recent study showing the RDR6-dependent production of phased and unphased siRNAs from RNAs that do not appear to be targeted by siRNAs or miRNAs [45].

In *Arabidopsis*, RDR2 and RDR6 are involved in the transcription of RNA targeted by primary siRNA or miRNA leading to production of secondary siRNAs [1–5,19,20]. One possible mechanism for the synthesis of the complementary strand 5’ of the smRNA binding site is the elongation of smRNA hybridized to the target RNA [1,19,20,46,47]. Indeed *in vivo* evidence for smRNA elongation was provided in the study of the RDR6-dependent silencing of a miR171(21nt)-sensor transgene in *Arabidopsis* [20]. For RDR2-dependent secondary siRNA synthesis, it has been shown using a transgene reporter system that the silencer locus produced primary siRNAs of 21-, 22- and 24-nt [19]. It remains to be determined whether RDR2 recruitment is dependent on the size of the primary siRNA, and to date there is no *in vivo* evidence of an RDR2 priming activity. Our finding that RDR2-HA and RDR6-HA can elongate self-primed ssRNA templates prompted us to assess their ability to elongate smRNA hybridized to ssRNA templates and to test if this ability was smRNA-size dependent. RDR2-HA was able to fully elongate the template with no preference for smRNA size (Fig. 4A and 4C). RDR6-HA was also able to perform smRNA-primed elongation although with a lower efficiency (Fig. 4B and 4C). These results show that RDR2 and RDR6 have an intrinsic capacity to elongate smRNA *in vitro*, and that this activity is not dependent on the length of the smRNA *in vitro*. Priming activity was not detected in previous *in vitro* studies with recombinant HA-RDR6 [29,30] however different HA tag locations were used (at the C-terminus in our work and at the N-terminus previously). Furthermore, we show that the NTPs concentration in the reaction buffer has a crucial influence on priming activities, and that priming activity is strongly suppressed at the concentrations used in the previous studies (Fig. 4C). Although detectable, we found that *in vitro* RDR6-HA priming was weak, and we suggest that this is due to the absence of cellular co-factors that promote priming *in vivo*. One of these co-factors could be the dsRNA-binding protein SGS3 that physically interacts with RDR6 [40] and stabilizes RDR6 templates *in vivo* [15]. Furthermore, *in vitro* SGS3 can bind to partially dsRNA with a 5’ overhang [30] that could mimic the *in vivo* hybridization of an siRNA or miRNA to a target RNA. Similarly, the SGS3-like protein RDM12, involved in RNA-directed DNA methylation and transcriptional gene silencing can be a co-factor of RDR2 [48]. The experimental set up described in the present work will allow the assessment of the roles of SGS3 and RDM12 in modulating RDR6 and RDR2 polymerase activities and particularly their priming activity. To date there is no evidence for an *in vivo* priming activity of RDR2. Our results clearly show that RDR2 has the intrinsic ability to initiate primed RNA synthesis thus opening up a new field of investigation into the mechanism of RDR2-dependent RNA silencing.

## Supporting Information

S1 Fig Polymerisation activity of RDR2-HA and RDR6-HA on an ssRNA template. Purified RDR2-HA and RDR6-HA were incubated with cold NTPs and 5'[32P]-labeled 37-nt RNA template (RNA37A☢). In these conditions, detection of labeled products higher than the template size (>37nt, higher products) correspond only to elongated template. (*) A single star indicates the position of the RNA37A☢ template.
(TIF)

S2 Fig Polymerisation activity of RDR2-HA and RDR6-HA on ssRNA template RNA38. RNA38 is designed to adopt a stable hairpin-like conformation in the 3' terminus region. Purified RDR2-HA and RDR6-HA were incubated with 5'[32P]-labeled 38-nt ssRNA template (RNA38☢), cold NTPs and trace amounts of [α32-GTP]. (*) A single star indicates the position of the RNA38☢ template.
(TIF)

S3 Fig Nucleotide sequences of partial dsRNA templates used in RDR2-HA and RDR6-HA priming assays. RNA37A template was hybridized to either 5‘[32P]-labeled RNA of 21- (RNA21☢), 22- (RNA22☢) or 24-nt (RNA24☢) sizes oligonucleotides.
(TIF)

S4 Fig Purified RDR2-HA and RDR6-HA have priming activities on partial dsRNA templates. Purified RDR2-HA and RDR6-HA were incubated with partial dsRNAs formed with RNA37A hybridized with 5’[32P]-labeled 22-nt ssRNA (RNA22☢). 5'[32P]-labeled RNA37A (RNA37A☢) and 5'[32P]-labeled RNA22 (RNA22☢) are used as a size markers. (*) Simple star indicate the position of the RNA22☢. (**) Double star indicates the position of the full-length elongated RNA template.
(TIF)

S1 Table Primers sequences (5’-3’). (PDF)

S2 Table RNA templates sequences (5’-3’). (PDF)
