# Supplementary figures and images for: Primer-Dependent and Primer-Independent Initiation of Double Stranded RNA Synthesis by Purified Arabidopsis RNA-Dependent RNA Polymerases RDR2 and RDR6
Source: PLoS One. 2015 Mar 20;10(3):e0120100. doi: 10.1371/journal.pone.0120100 (PMC4368572; doi:10.1371/journal.pone.0120100)

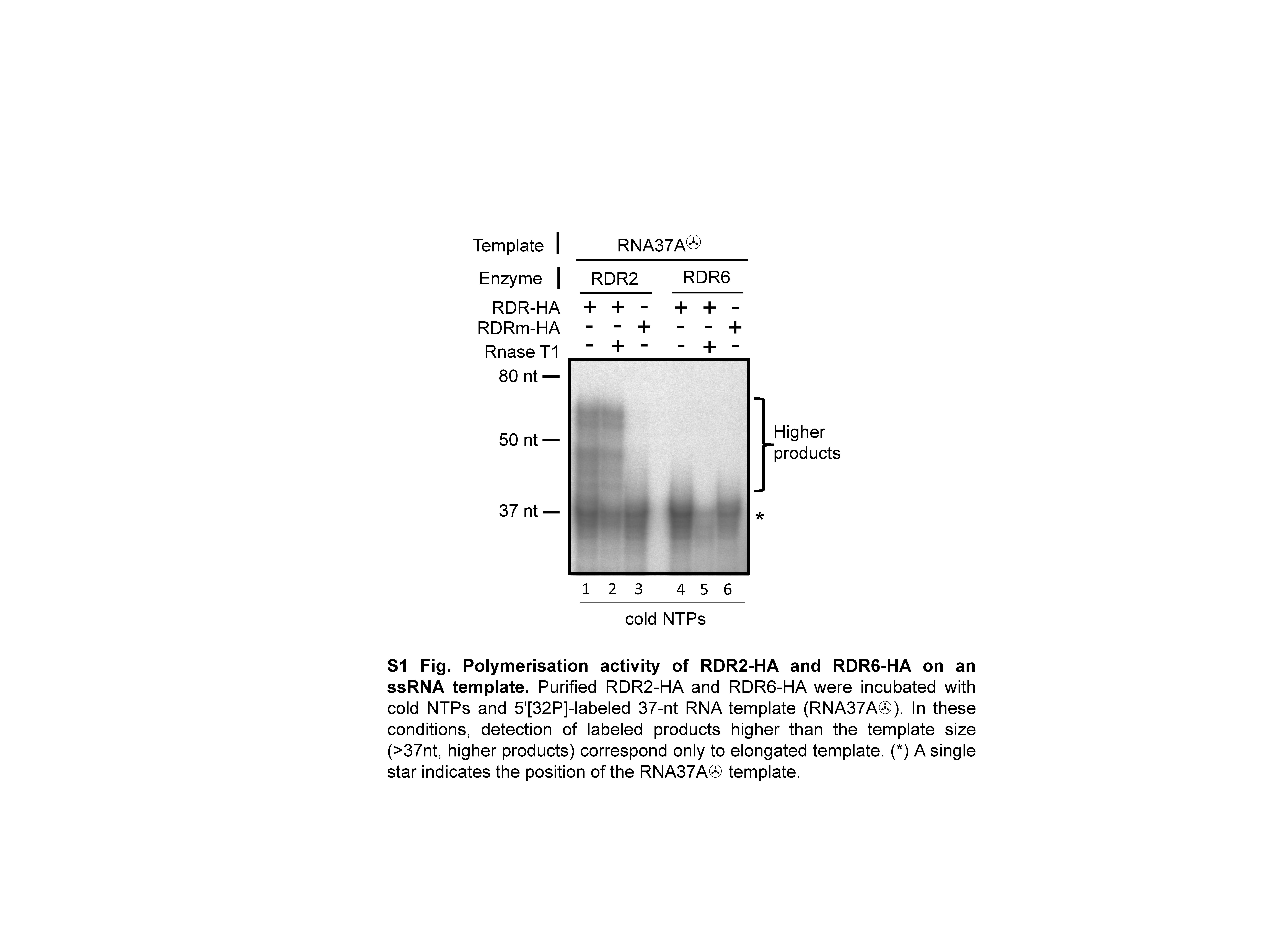

Supplement: S1 Fig — Purified RDR2-HA and RDR6-HA were incubated with cold NTPs and 5'[32P]-labeled 37-nt RNA template (RNA37A☢). In these conditions, detection of labeled products higher than the template size (>37nt, higher products) correspond only to elongated template. (*) A single star indicates the position of the RNA37A☢ template. (TIF) [file pone.0120100.s001.tif]

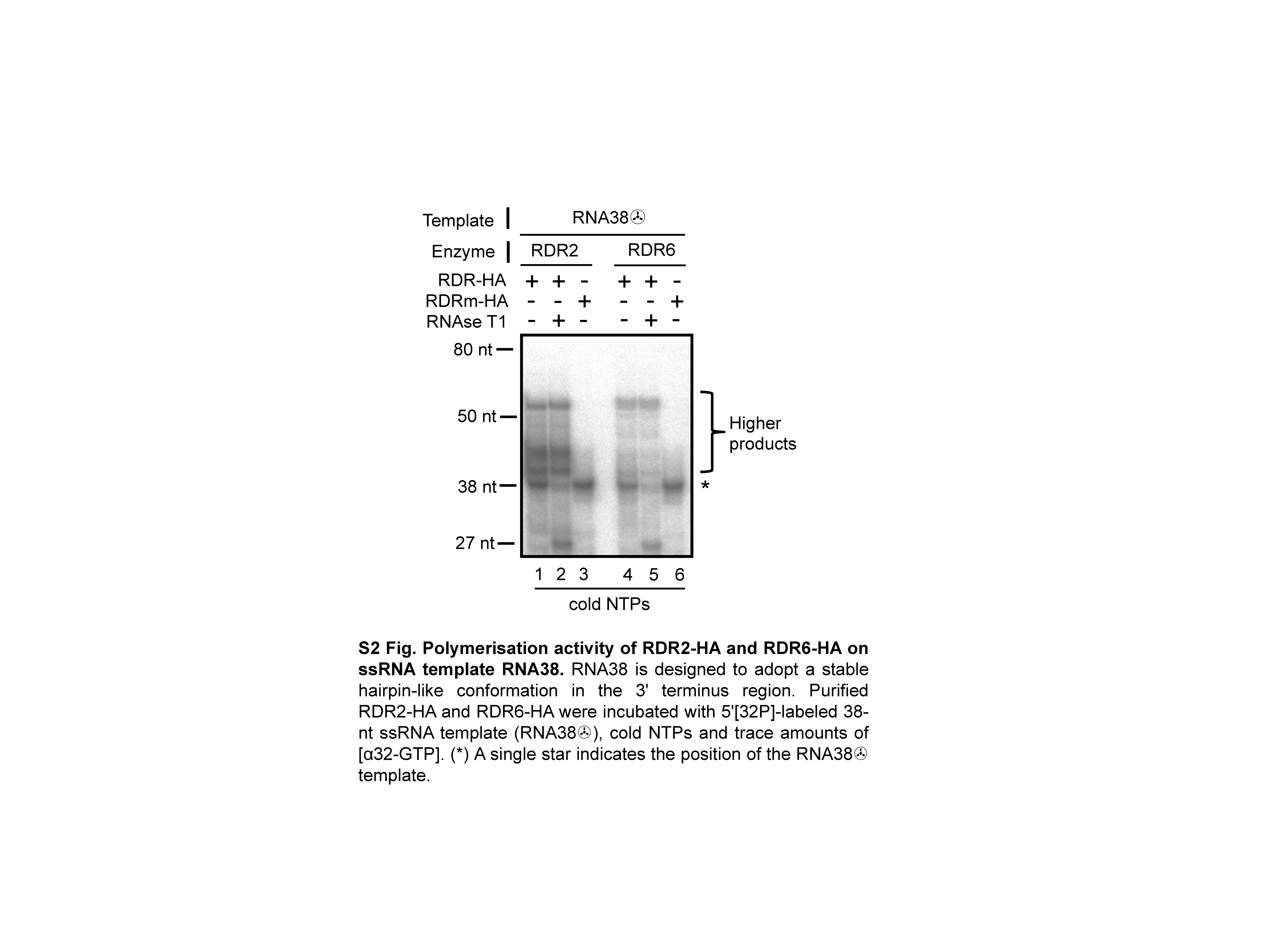

Supplement: S2 Fig — RNA38 is designed to adopt a stable hairpin-like conformation in the 3' terminus region. Purified RDR2-HA and RDR6-HA were incubated with 5'[32P]-labeled 38-nt ssRNA template (RNA38☢), cold NTPs and trace amounts of [α32-GTP]. (*) A single star indicates the position of the RNA38☢ template. (TIF) [file pone.0120100.s002.tif]

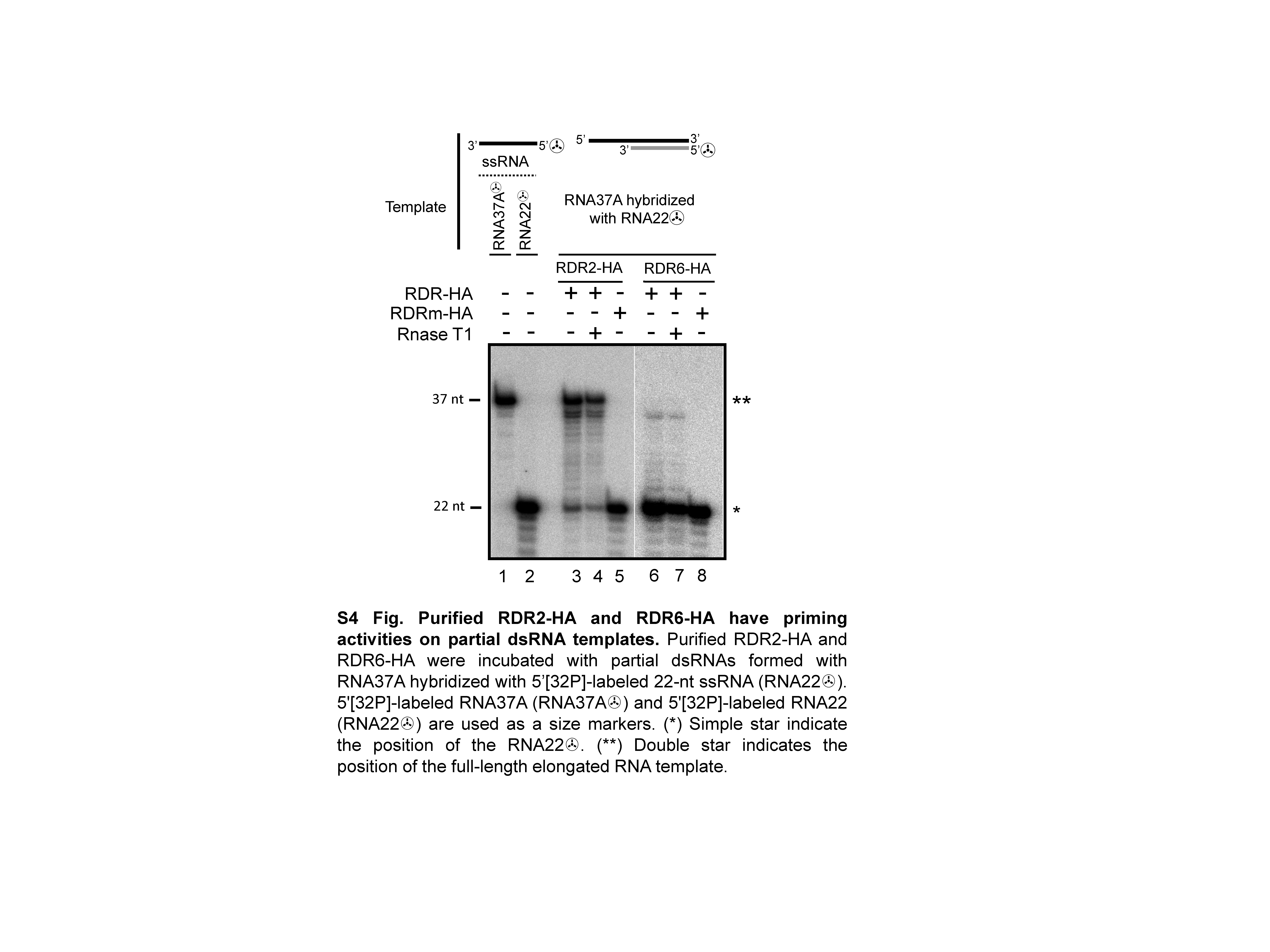

Supplement: S4 Fig — Purified RDR2-HA and RDR6-HA were incubated with partial dsRNAs formed with RNA37A hybridized with 5’[32P]-labeled 22-nt ssRNA (RNA22☢). 5'[32P]-labeled RNA37A (RNA37A☢) and 5'[32P]-labeled RNA22 (RNA22☢) are used as a size markers. (*) Simple star indicate the position of the RNA22☢. (**) Double star indicates the position of the full-length elongated RNA template. (TIF) [file pone.0120100.s004.tif]
