# Supplementary material for: Primer-Dependent and Primer-Independent Initiation of Double Stranded RNA Synthesis by Purified Arabidopsis RNA-Dependent RNA Polymerases RDR2 and RDR6
Source: PLoS One. 2015 Mar 20;10(3):e0120100. doi: 10.1371/journal.pone.0120100 (PMC4368572; doi:10.1371/journal.pone.0120100)
Supplement: S1 Table — (PDF) [file pone.0120100.s005.pdf]

S1 Table. Primers sequences (5’-3’).

AttB1-RDR2	GGGGACAAGTTTGTACAAAAAAGCAGGCTTGatggtgtcagagacgacgacgaaccgatc
AttB2-RDR2	GGGGACCACTTTGTACAAGAAAGCTGGGTCaatggatacaagtccacttgttttctcttc
AttB1-RDR6	GGGGACAAGTTTGTACAAAAAAGCAGGCTTGatggggtcagagggaaatatgaagaagtc
AttB2-RDR6	GGGGACCACTTTGTACAAGAAAGCTGGGTCcgagacgctgagcagaaacttagccaaag
Age1-RDR2	cgcgtaccggtATGGTGTCAGAGACGACGACGAACCG
SmaI-RDR2-2xHA	tgattcccgggTTAagcgtaatctggaacatcgtatgggtaagcgtaatctggaacatcgtatgggtaAATGGATACAAGTCCACTTGTTTTCTCTTCA
AgeI-RDR6	tcgcgaccggtATGGGGTCAGAGGGAAATATGA
XhoI-2xHA-RDR6	aggcctcgagTTATTAagcgtaatctggaacatcgtatgggtaagcgtaatctggaacatcgtatgggtaGAGACGCTGAGCAAGAAAC
RDR2mutFW	GTTCTGGTGGCGATCTCGACGGAGCCCAGTTTTTTGTTAGCTGGGATGAGAAG
RDR6mutFW	GACCTTGACGGGGCCCTGTACTTTGTGGCTTGGGATCAGAAACTCATCCTC
