# Supplementary material for: Primer-Dependent and Primer-Independent Initiation of Double Stranded RNA Synthesis by Purified Arabidopsis RNA-Dependent RNA Polymerases RDR2 and RDR6
Source: PLoS One. 2015 Mar 20;10(3):e0120100. doi: 10.1371/journal.pone.0120100 (PMC4368572; doi:10.1371/journal.pone.0120100)
Supplement: S2 Table — (PDF) [file pone.0120100.s006.pdf]

S2 Table. RNA templates sequences (5’-3’).

RNA37A	pAAUCGUGAACUUUCAAACUAUACAACCUACUACCUCA
RNA37U	pUGAGGUAGUAGGUUGUAUAGUUUGAAAGUUCACGAUU
RNA38	pUUCAAACUAUACAACCUACUACCUCAAGGCUUCAAGCC
RNA24	pUGAGGUAGUAGGUUGUAUAGUUUG
RNA22	pUGAGGUAGUAGGUUGUAUAGUU
RNA21	pUGAGGUAGUAGGUUGUAUAGU
RNA22ddC	pUGAGGUAGUAGGUUGUAUAGUddC
